# Interactive analysis of large cancer copy number studies with Copy Number Explorer

**DOI:** 10.1093/bioinformatics/btv298

**Published:** 2015-05-07

**Authors:** Scott Newman

**Affiliations:** Biostatistics & Bioinformatics Shared Resource, Winship Cancer Institute of Emory University, Atlanta, GA 30322, USA

## Abstract

**Summary:** Copy number abnormalities (CNAs) such as somatically-acquired chromosomal deletions and duplications drive the development of cancer. As individual tumor genomes can contain tens or even hundreds of large and/or focal CNAs, a major difficulty is differentiating between important, recurrent pathogenic changes and benign changes unrelated to the subject’s phenotype. Here we present Copy Number Explorer, an interactive tool for mining large copy number datasets. Copy Number Explorer facilitates rapid visual and statistical identification of recurrent regions of gain or loss, identifies the genes most likely to drive CNA formation using the cghMCR method and identifies recurrently broken genes that may be disrupted or fused. The software also allows users to identify recurrent CNA regions that may be associated with differential survival.

**Availability and Implementation:** Copy Number Explorer is available under the GNU public license (GPL-3). Source code is available at: https://sourceforge.net/projects/copynumberexplorer/

**Contact:** scott.newman@emory.edu

## 1 Motivation

Huge volumes of genomics data from nearly every cancer type are now freely available and innovative projects such as UCSC Cancer Genomics Browser (Cline *et al.*, 2013), cBioPortal (Cerami *et al.*, 2012) and arrayMap (Cai *et al*., 2015) have begun to collate and store this information.

Those analyzing cancer copy number data often wish to know the location of recurrent CNAs in a given tumor type and which genes are likely to be driving that gain or loss. Additionally, some may wish to know which genes are recurrently broken as this can indicate a potential tumor suppressor loss or gene fusion. It is, however, challenging to analyze CNA regions in these ways using current gene-centric or non-interactive approaches. This is problematic since the presence or absence of a certain CNA can aid in diagnosis, stratify patients into different risk categories or inform therapy decisions.

Copy Number Explorer rapidly generates and displays interactive CNA and breakage frequency plots from public data such as the TCGA or from the user's own study. The software requires only segmented copy number data as is generated from popular segmentation algorithms such as DNAcopy (Venkatraman and Olshen, 2007). Subsequently, it is possible to combine data from different studies or from an internal database—even if data were generated using different array designs or next generation sequencing. Here we demonstrate the utility of Copy Number Explorer by applying it to the TCGA Glioblastoma Multiforme (GBM) data (Brennan *et al.*, 2013).

## 2 Implementation

The software is written in R and tested using version 3.0.2. Tier 3 segmented SNP6 array copy number and survival data were downloaded from the TCGA data portal (https://tcgadata.nci.nih.gov-/tcga/). The software calculates CNA frequency of every gene in the human hg19 RefSeq gene set by assigning it the log2 ratio of the copy number segment from which it was derived. By default, any gene whose segmented log ratio is greater than 0.2 is considered gained and less than −0.2 is considered lost (thresholds are, however, customizable) and the total number of samples crossing these thresholds are calculated. Segment Gain or Loss (SGOL) scores are calculated for RefSeq genes using the SGOL function of the cghMCR package (http://bioconductor.org/packages/release-/bioc/ html/cghMCR.html). Gene-level frequency of gain, loss and breakage are calculated using Perl and R scripts. Survival analysis is performed with the R survival package according to standard methods. Interactive CNA plots and survival curves are made visible through a web browser using the Shiny R package (www.rstudio.com/shiny). An online version was deployed using ShinyApps.io (www.shinyapps.io).

## 3 Example

**Fig. 1.**
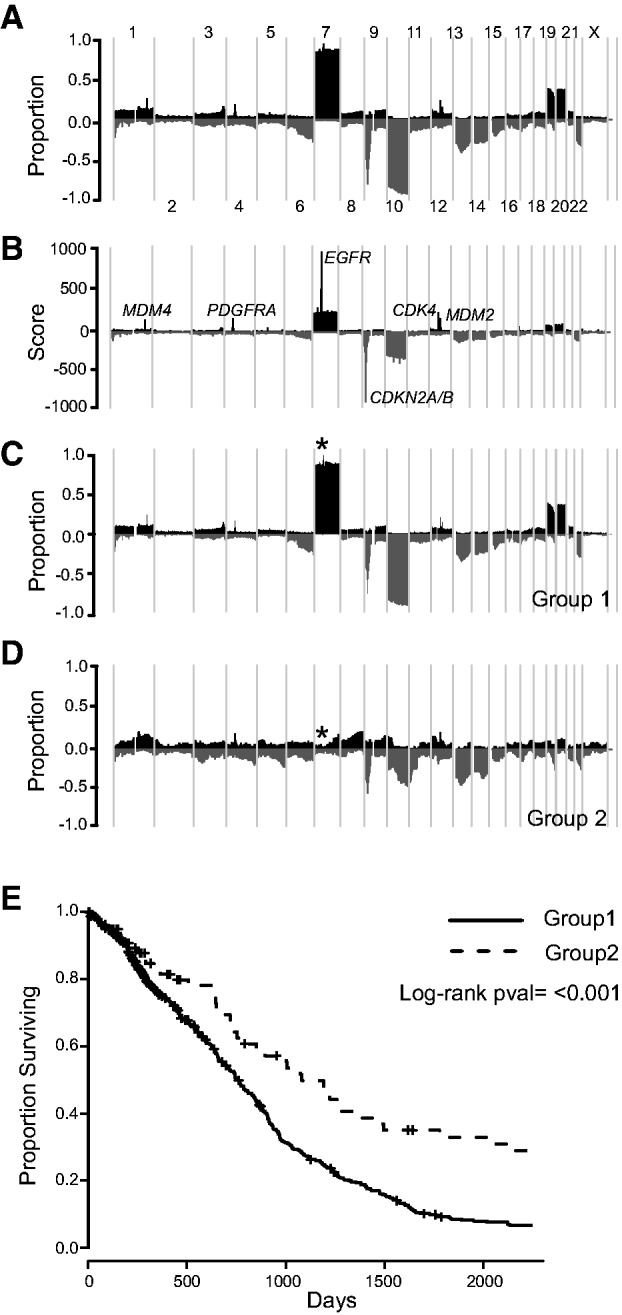
Copy Number Explorer Workflow. (A) Aberration frequency plot shows proportion of samples with gains and losses from GBM (*n* = 580). Chromosomes are placed in order along the *x*-axis and the proportion of gains (dark grey; positive numbers) or losses (light grey; negative numbers) are shown on the y-axis. (B) cghMCR-transformed data results in a segment gain or loss (SGOL) score on the *y*-axis. High absolute SGOL scores indicate one or a combination of highly focal, high frequency or a high magnitude of alterations across multiple samples: SGOL score can be used to identify the gene most likely to be the target of gain or loss. Genes with the highest SGOL scores for chromosomes 1, 4, 7, 9 and 12 are indicated. (C, D) Data is partitioned based on the presence of a gain or amplification of the *EGFR* locus (indicated by an asterisk). Group 1 (*n* = 499) (C) shows 100% of cases have this abnormality whereas no cases in Group 2 (n = 81) do (D). (E) Kaplan–Meier curve shows that subjects with an *EGFR* gain or amplification have a poorer prognosis than those without

Copy Number Explorer produces frequency profiles in which recurrent CNA regions can be identified. For example, the combined profile from 580 GBM tumors showed gains of chromosome seven and losses of chromosome ten were the most common events (Fig. 1A). Numerous other focally altered (<∼10 Mb) regions were also evident including loss of 9p21.3 containing *CDKN2A/B* in ∼70% of samples and amplification or gain 1q32.1 containing *MDM4* and *PIK3C2B* in ∼23% of samples. These findings are in keeping with other published studies (Brennan *et al.*, 2013).

Several statistical frameworks aim to identify the gene targets of amplification and deletion including GISTIC, RAE and cghMCR (Aguirre *et al.*, 2004; Beroukhim *et al.*, 2007; Taylor *et al.*, 2008). Copy Number Explorer deploys cghMCR as it runs within R and does not require raw data or probe mapping information. The cghMCR transformation exacerbated known recurrent abnormalities such as amplifications of *MDM4*, *PDGFR*, *EGFR*, *CDK4* and *MDM2* and deletions of *CDKN2A/B* (Fig. 1B).

The presence of a CNA, such as *EGFR* amplification in GBM, can predict outcome (Verhaak *et al.*, 2010). Copy Number Explorer can partition the data based on the presence of a gain, amplification or homozygous loss in any genomic region. If the dataset is clinically annotated, then the software can construct Kaplan–Meier curves for each group that are compared based on a log-rank test. For example, after partitioning the data based on the presence of gain or amplification of any region containing *EGFR* (chr7:55086725-55275031[hg19]) the gained/amplified group (*n* = 499) showed a poorer prognosis than the non-gained/amplified group (*n* = 81) (*P* < 0.0001 by log rank test) (Fig. 1C–E).

Copy number change points often fall within genes and imply disruption due to a structural change such as an unbalanced translocation. Gene breakage can imply loss of function or alternatively gain of function through gene fusion. Copy Number Explorer produces breakpoint frequency plots summarizing the proportion of cases with copy number change points within each gene. The three most broken genes in the TCGA data were *EGFR* (27%), *CDKN2B-AS1* (19%) and *PTEN* (8%; not shown). Breakage of the *EGFR* gene may imply an *EGFR* gene fusion as has been described previously while breaks in *CDKN2B-AS1* and *PTEN* imply disruption of tumor suppressor genes either at or close to the break (Brennan *et al.*, 2013).

## 4 Conclusions

Copy Number Explorer is a free and easy to use tool that aids in the interpretation of copy number data from large cancer studies.

We used the TCGA GBM data for this analysis, but the software can use segmented copy number data from any source, indeed the online version currently houses all publically released TCGA copy number datasets. We also note that a private instance can be run on the user's own local computer or easily be deployed online with a single R command using ShinyApps.io.
